# Meta-analyses of the proportion of Japanese encephalitis virus infection in vectors and vertebrate hosts

**DOI:** 10.1186/s13071-017-2354-7

**Published:** 2017-09-07

**Authors:** Ana R.S. Oliveira, Lee W. Cohnstaedt, Erin Strathe, Luciana Etcheverry Hernández, D. Scott McVey, José Piaggio, Natalia Cernicchiaro

**Affiliations:** 1 Department of Diagnostic Medicine and Pathobiology, College of Veterinary Medicine, Kansas State University, Manhattan, KS USA; 2 United States Department of Agriculture-Agricultural Research Service (USDA-ARS) Arthropod-Borne Animal Diseases Research, 1515 College Ave., Manhattan, KS USA; 3 Department of Clinical Sciences, College of Veterinary Medicine, Kansas State University, Manhattan, KS USA; 4 School of Veterinary Medicine, University of the Republic, Montevideo, Uruguay

**Keywords:** Japanese encephalitis virus, Japanese encephalitis, Meta-analysis, Vector, Host, Competence

## Abstract

**Background:**

Japanese encephalitis (JE) is a zoonosis in Southeast Asia vectored by mosquitoes infected with the Japanese encephalitis virus (JEV). Japanese encephalitis is considered an emerging exotic infectious disease with potential for introduction in currently JEV-free countries. Pigs and ardeid birds are reservoir hosts and play a major role on the transmission dynamics of the disease. The objective of the study was to quantitatively summarize the proportion of JEV infection in vectors and vertebrate hosts from data pertaining to observational studies obtained in a systematic review of the literature on vector and host competence for JEV, using meta-analyses.

**Methods:**

Data gathered in this study pertained to three outcomes: proportion of JEV infection in vectors, proportion of JEV infection in vertebrate hosts, and minimum infection rate (MIR) in vectors. Random-effects subgroup meta-analysis models were fitted by species (mosquito or vertebrate host species) to estimate pooled summary measures, as well as to compute the variance between studies. Meta-regression models were fitted to assess the association between different predictors and the outcomes of interest and to identify sources of heterogeneity among studies. Predictors included in all models were mosquito/vertebrate host species, diagnostic methods, mosquito capture methods, season, country/region, age category, and number of mosquitos per pool.

**Results:**

Mosquito species, diagnostic method, country, and capture method represented important sources of heterogeneity associated with the proportion of JEV infection; host species and region were considered sources of heterogeneity associated with the proportion of JEV infection in hosts; and diagnostic and mosquito capture methods were deemed important contributors of heterogeneity for the MIR outcome.

**Conclusions:**

Our findings provide reference pooled summary estimates of vector competence for JEV for some mosquito species, as well as of sources of variability for these outcomes. Moreover, this work provides useful guidelines when interpreting vector and host infection proportions or prevalence from observational studies, and contributes to further our understanding of vector and vertebrate host competence for JEV, elucidating information on the relative importance of vectors and hosts on JEV introduction and transmission.

**Electronic supplementary material:**

The online version of this article (10.1186/s13071-017-2354-7) contains supplementary material, which is available to authorized users.

## Background

Japanese encephalitis virus (JEV) is the causative agent of Japanese encephalitis (JE), the most important viral encephalitis occurring in humans, particularly in children aged 0–14 years, in Southeastern Asia and the Pacific Rim [1]. There are approximately 67,900 new JE cases every year (overall incidence 1.8 per 100,000 people) [1]. Japanese encephalitis is a mosquito-borne disease, with JEV being vectored by different species of mosquitoes, although the *Culex vishnui* subgroup, and particularly *Culex tritaeniorhynchus*, are considered the most relevant vectors [2].

The disease symptoms range from a nonspecific febrile illness to aseptic meningitis and severe encephalitis that may lead to irreversible neurological sequelae. Despite less than 1% of humans infected with JEV develop clinical disease, 20–30% of those cases are fatal, and about 30–50% of survivors experience neurological damage [1, 3].

Japanese encephalitis is more prevalent in rural and suburban areas, where both rice and pig production are present, as rice paddy fields provide the ideal conditions for mosquito breeding and pigs are considered the main amplifying reservoir for JEV [1, 4].

There are two main JEV transmission cycles, a pig-associated rural domestic and a bird-associated wild cycle. Although humans and domestic animals other than pigs and birds may become infected via infected mosquito bites, they do not develop sufficient viremia to infect susceptible vectors and they are thus considered dead-end hosts [4]. Japanese encephalitis transmission is highly dynamic, usually occurring in epidemics, especially in the most temperate regions of Asia (higher latitudes), while in the tropics and subtropics the disease is endemic, peaking in the rainy season [1].

Japanese encephalitis virus has spread to new regions over the past decades, reaching Pakistan in the west and the Torres Strait in Australia in the southeast. The recent expansion is not fully understood, although they may include inadvertent transportation of infected vectors, human movement, bird migration (with climate change affecting migration patterns), and wind-blown mosquitoes [5, 6]. Because expansion has occurred, there is a global concern related to the emergence of exotic vector-borne diseases, such as JE, in currently virus-free countries. Previous authors [7] reported that there is a considerable range of mosquito species that are susceptible to JEV and that, if competent vectors and vertebrate hosts are present in these regions, virus introduction is possible. Furthermore, other authors claimed that JEV viremia has been observed in more than 90 wild and domestic birds, belonging to several avian families, and that JEV has been isolated in over 30 mosquito species [2, 7]. Conversely, and despite many reviews and several references to the potential spread of JEV and the importance attributed to vector and vertebrate host competence in its introduction and transmission into new geographical areas, no quantification or thorough analysis of such parameters have been conducted so far [2, 7–9]. An accurate evaluation of such parameters would further our understanding of the relative importance of vectors and vertebrate hosts on virus introduction and transmission, ultimately pointing to more effective prevention and mitigation strategies [10].

A systematic review of the literature is a tool that allows a rigorous and consistent identification, assessment, and summary of current scientific evidence, whereas a meta-analysis is a quantitative, statistical method that combines the results of the data gathered from the body of evidence, providing a more accurate estimate (i.e. a summary effect measure) of the outcomes of interest. Moreover, a meta-analysis allows exploring the sources of heterogeneity between results from different studies, thus considering possible sources of confounding and bias. A meta-analysis increases the power of a systematic review and provides valuable information to answer the research question posed and/or identifies potential knowledge gaps [11–15].

Hence, the objective of this study is to quantitatively summarize the proportion of viral infection in vectors and vertebrate hosts from data pertaining to observational studies obtained in a systematic review of the literature on vector and host competence for JEV, using meta-analyses.

## Methods

### Systematic review of the literature

The literature search was conducted in eight electronic databases and journal websites (Web of Science, Pubmed, Armed Forces Pest Management Board, The American Journal of Tropical Medicine and Hygiene, Journal of Medical Entomology, Journal of the American Mosquito Control Association, Vector Borne and Zoonotic Diseases and Google Scholar) between March 28th, 2016 and April 25th, 2016. Additionally, a hand-search of the reference list of nine articles considered as key publications by the reviewers was performed [2–8, 16, 17].

The identified articles were screened for relevance following a set of inclusion and exclusion criteria (available as supplementary information in Additional file 1). To be considered relevant, the original research article (no literature searches or reviews were included) had to be written in English language and peer-reviewed. No restrictions regarding time of publication or geographical area were imposed. Outcome measures of interest included vector transmission efficiency (infection, dissemination, and transmission rates), host preference of vectors, and susceptibility to infection (minimum infection rates, maximum likelihood estimator for vectors, and proportion of JEV infection for both vectors and host species). Relevance screening was performed by two reviewers (AO, LH) working independently and conflicts were resolved by consensus or by consulting a third reviewer whenever consensus could not be reached.

Data pertaining to the outcome measures previously identified were then retrieved to an Excel® (Microsoft Corp., Redmond WA, 2013) spreadsheet, using a pre-designed template. Information on internal and external validity of the articles (assessment of the risk of bias) was evaluated by two reviewers (AO, NC) working independently in a set of 10 articles, with all remaining articles being assessed by one reviewer. Assessment of the risk of bias was based on a set of criteria related to the study question, study population, inclusion and exclusion criteria, study period, study area, exposures, outcomes, and bias, for observational studies; and study question, study population, intervention, experimental conditions, experimental setting, randomization, blinding, and outcomes, for experimental studies.

We followed the protocols described by Sargeant & O’Connor [15, 18], and O’Connor et al. [14, 19] for performing systematic reviews in veterinary medicine, and the Cochrane Review Handbook guidelines [20] for the risk of bias assessment.

Information regarding all steps of the systematic review, including a complete list of search terms, summary of search results, inclusion and exclusion criteria, outcomes, and identification of key domains for risk of bias assessment in observational and experimental studies) are available as supplementary information (Additional file 1).

### Data analysis

#### Meta-analysis

To assess vector and host competence for JEV, we performed meta-analyses for three outcomes whose data we gathered from observational studies: proportion of JEV infection in vectors, proportion of JEV infection in vertebrate hosts, and minimum infection rate (MIR) in vectors. We did not carry out a meta-analysis for maximum likelihood estimator (MLE), though it was an outcome included in the systematic review, as the data pertained to very few articles (*n* = 6).

The definition of the outcomes of interest is available in Table 1. Proportion of JEV infection was computed as the number of positive units (mosquito pools or vertebrate hosts) divided by the total number of sampled units and it expresses a probability. Minimum infection rate was defined as the ratio of the number of positive mosquito pools to the total number of mosquitoes in the sample, assuming that only one infected individual is present in a positive pool [21].

**Table 1:** Outcome measures quantified in the meta-analyses

	Vector competence	Host competence
Susceptibility to infection	Proportion of JEV infection^a^	Proportion of JEV infection^b^
	Minimum infection rate^c^	–
	Maximum likelihood estimator^d^	–

^a^Proportion of JEV infection is the sum of positive mosquito pools divided by the total number of pools tested in observational studies

^b^Proportion of positive vertebrate hosts equals the sum of positive samples divided by the sum of samples tested

^c^Minimum infection rate (MIR) is defined as the ratio of the number of positive mosquito pools to the total number of mosquitoes in the sample, assuming that only one infected individual is present in a positive pool [21]

^d^Maximum likelihood estimator (MLE) represents the proportion of infected mosquitoes that maximizes the likelihood of the number of pools of a specific size to be virus positive, where the proportion is the parameter of a binomial distribution [21]

Articles reporting only the total percentage of infection (or MIR), without specifying the numerator or denominator, were not considered in this meta-analysis. Similarly, articles reporting only the number of positive samples or the total number of samples were not considered.

For the meta-analysis of the proportion of JEV infection in vectors, we only included articles reporting mosquito species with more than 1% infection and more than 1000 individual mosquitoes. Articles describing percentages of infection and the overall total number of mosquitoes tested without describing specific pool sizes were still included. For the purposes of this study, we assumed these pools contained at least 1000 individual mosquitoes. For the outcomes proportion of JEV infection in vertebrate hosts and MIR in vectors, all observations pertaining to all host and mosquito species reported were included.

Proportions and MIR reported were first logit-transformed and standard errors (S.E.) of the logit of the proportion or MIR were then computed, according to the following formulae [22]:

$$ \mathrm{logit}\  \mathrm{proportion}=\ln \left(\frac{p}{1-p}\right)\kern0.5em \mathrm{S}.\mathrm{E}.=\sqrt{\frac{1}{n\times p\times \left(1-p\right)\ }} $$

where *p* is the proportion of JEV infection or MIR and *n* is the sample size (i.e. total number of sampled units - mosquito pools, vertebrate hosts, or individual mosquitoes). For interpretation, the pooled logit estimates and their 95% confidence intervals were back-transformed [23], as follows:

$$ p=\frac{e^{logit}\ }{e^{logit}+1} $$

Whereas the data inputted in the models corresponded to proportions, estimates from the meta-analysis (i.e. pooled logit estimates) correspond to percentages.

We assumed that substantial heterogeneity existed among the studies, hence, we decided a priori to fit a random-effects meta-analysis using the method of DerSimonian & Laird [24] to estimate the variance between studies, using a restricted maximum likelihood (REML) algorithm. Moreover, subgroup meta-analyses by species (mosquito species or vertebrate host) were performed by running independent models for the three different outcomes using the *metan* command in Stata-SE 12.0 (StataCorp., College Station TX, USA).

#### Meta-regression

Meta-regression models were fitted to identify sources of heterogeneity among studies and to assess the association between different predictors and the outcomes of interest: proportion of JEV infection in vectors, proportion of JEV infection in vertebrate hosts and MIR (defined above).

Random effects meta-regression models using the restricted maximum likelihood method [*metareg*, Stata-SE 12.0 (StataCorp., College Station TX, USA)] were performed following the formula:

$$ {\mathrm{logit}\  \mathrm{proportion}}_j={\beta}_0+\beta {X}_j+{\mu}_j+{\varepsilon}_j $$

where *β*_0_ is the intercept, *βX*_*j*_ is the coefficient for the *j*th predictor, *μ*_*j*_ is the effect of study *j*, and *ε*_*j*_ is the error term, i.e. the differences between studies due to sampling variation. Meta-regression models were performed using logit transformed outcomes (and expressed as percentages) and within-study standard errors.

We quantified heterogeneity using the *I* ^2^ value, which represents the proportion of total variability in pooled estimates that can be attributed to heterogeneity [19], and followed the recommendations for interpretation given by O’Connor et al. [19]: *I* ^2^ values of 0–40%: unimportant heterogeneity; 30–60%: moderate heterogeneity; 50–90%: substantial heterogeneity; and 75–100%: considerable heterogeneity.

To evaluate predictors that may have contributed to the variation in results across studies, we fitted univariable meta-regression models (testing one predictor at a time) followed by a multivariable model (testing multiple predictors simultaneously). Univariable analyses between predictors of interest (i.e. mosquito or vertebrate host species) and outcomes were performed and their significance assessed using a partial *F*-test; *P*-values < 0.1 were deemed significant and used to determine the inclusion of the predictors in the multivariable main effects model.

Predictors of interest for the outcome pertaining to the proportion of JEV infection in vectors included mosquito species, diagnostic method, country, capture method, season, and number of mosquitoes per pool. For the outcome related to the proportion of JEV infection in hosts, predictors included host species, region, season, age category, and diagnostic method. Predictors for the MIR outcome included mosquito species, diagnostic method, capture method, season, and country.

Confounding was assessed by including each predictor, considered as a priori confounder based on causal diagrams, in the model at a time (bivariable analysis) and checking for changes in the coefficients, both in magnitude (> 30%) and direction, and changes in *P*-values of the main predictors of interest.

If there was evidence of confounding, the confounder was kept in the model. If there was no evidence of confounding and the predictor was no longer significant (*P*-value > 0.1), it was removed from the final model.

Whenever there were concerns of overfitting the model, which can affect the precision of the parameter estimates and test statistics, we present the results from univariable analyses. The dataset had to contain a minimum of 10 (*k* + 1) observations, where *k* is the number of predictors in the model, in order to adequately fit the model, following Hosmer & Lemeshow’s recommendations [25].

For the outcome proportion of JEV infection in vectors, a second model was performed including only the mosquito species represented in more than 10 articles. The same procedures, regarding univariable and multivariable analyses and assessment of confounding, were followed.

#### Predictors and outcomes

Table 2 provides a detailed description of the predictors included in the meta-regression analyses, which were selected for inclusion based on biological importance and completeness of observations. Mosquito species included different genera and/or species. The variable pertaining to vertebrate hosts was categorized as follows: pigs, birds, sylvatic mammals, cattle, sheep and goats, cats and dogs, chickens, ducks, rabbits, herons, horses and donkeys, wild pigs, bats, rats, reptiles and amphibians, based on how the data were presented in the relevant studies and/or number of observations in each category. Season was categorized into trimesters, so that trimester 1 included the months of December-February, trimester 2 included the months of March-May, trimester 3 June-August, and trimester 4 September-November. Two other categories were created, one referring to studies performed across the year (all year round), and the other when more than one trimester was recorded (more than one trimester).

**Table 2:** Predictors pertaining to study characteristics included in the meta-analyses of all outcomes

Variable	Description	Categories
Species	Mosquito/vertebrate host species or genera	Vectors: several species (*n* = 24); Hosts: pigs, birds, sylvatic mammals, cattle, sheep and goats, cats and dogs, chickens, ducks, rabbits, herons, horses and donkeys, wild pigs, bats, rats, reptiles and amphibians
Season	Trimester of the year during which the study was conducted	Trimester 1 (December-February), trimester 2 (March-May), trimester 3 (June-August), trimester 4 (September-November), all year-round
Diagnostic method	Diagnostic method used for detecting JEV	Vectors: virus isolation, antigen-capture enzyme assays, PCR^a^; Hosts: EIA or immunochromatography, hemagglutination inhibition tests, neutralization tests^b^
Capture method	Capture method used for capturing mosquitoes	Manual passive, manual active, mechanical visual, mechanical olfactory^c^
Mosquitoes/pool	Number of mosquitoes included in each pool	–
Country category	Country category where the study was conducted	Vectors: Australasia, India, China and Taiwan, Japan and South Korea, Thailand^d^; Hosts: North and South^e^
Age	Age of vertebrate host	Young and adult

^a^Virus isolation may use cell culture techniques or insect bioassays and virus identification by serotype identification with antibodies, such as indirect immunofluorescence assay (IFA)

Antigen-capture enzyme assays include the detection of antigens by enzyme immunoassays (EIA), alone or in combination with virus isolation. PCR or RT-PCR was used alone or in combination with antigen-capture enzyme assays, virus isolation, or both

^b^EIA or immunochromatography includes the detection of antibodies by EIA or immunochromatography only, or in combination with other methods, such as hemagglutination inhibition tests (HAI), virus isolation, and neutralization tests. Hemagglutination inhibition tests (HAI) may have been used alone or in combination with virus isolation and neutralization tests. Neutralization tests, including PRNT, may have been used alone or in combination with virus isolation. Virus isolation only is also included in this category

^c^Manual passive method includes aspirations; manual active uses sweep or drop nets; mechanical visual uses visual attractants, such as UV (black light) or white light; mechanical olfactory uses olfactory attractants, such as octanol

^d^Australasia includes Australia, Papua New Guinea, Indonesia and Saipan (Mariana islands); “India” includes India, Sri Lanka, and Bangladesh; “Thailand” includes Thailand, Malaysia and Vietnam

^e^North includes the following countries: China, Japan, and South Korea. South includes the following countries: Australia, Guam (USA), India, Myanmar, Nepal, Saipan (USA), Singapore, Sri Lanka, Taiwan, Thailand and Vietnam [26]

The variable corresponding to diagnostic methods used for diagnosis of JEV differed between vectors and vertebrate hosts. For vectors, diagnostic methods were classified into: (i) virus isolation methods [using cell culture techniques or insect bioassays and virus identification by serotype identification with antibodies, such as indirect immunofluorescence assay (IFA)]; (ii) antigen-capture enzyme assays (detection of antigens by enzyme immunoassays (EIA) (alone or in combination with virus isolation)); and (iii) PCR (PCR or RT-PCR alone or in combination with antigen-capture enzyme assays, virus isolation, or both). For vertebrate hosts, diagnostic methods were categorized as: (i) EIA or immunochromatography (detection of antibodies by EIA or immunochromatography only, or in combination with other methods, such as hemagglutination inhibition tests (HAI), virus isolation, and neutralization tests); (ii) hemagglutination inhibition tests (HAI) (HAI only, or in combination with virus isolation and neutralization tests); and (iii) neutralization tests (including plaque reduction neutralization test (PRNT), only, or in combination with virus isolation, and virus isolation only).

Mosquito capture methods were classified as: (i) manual passive (aspirations); (ii) manual active (use of sweep nets or drop nets); (iii) mechanical visual (use of visual attractants like UV (black light) or white light); (iv) mechanical olfactory (use of olfactory attractants like CO_2_ and other lures, such as octanol); (v) mechanical visual and olfactory (use of both visual and olfactory attractants); and (vi) manual and mechanical (any combination of manual and mechanical capture methods).

The variable pertaining to country of origin differed according to the outcome of interest and included one or more countries based on number of observations and geographical proximity. For outcomes related to vectors, categories for country included: (i) Australasia [including Australia, Papua New Guinea, Indonesia and Saipan (Mariana Islands)]; (ii) India (including India, Sri Lanka and Bangladesh); (iii) China and Taiwan, Japan and South Korea; and (iv) Thailand (Thailand, Malaysia and Vietnam). The rationale used for determining these categories was geographical proximity of the countries reported in the articles and number of observations each contained. For outcomes related to vertebrate hosts, we considered two categories for region/country of origin: (i) North (including China, Japan and South Korea); and (ii) South [including Nepal, Taiwan, India, South Korea, USA, Japan, China, Thailand, Sri Lanka, Myanmar, Vietnam, Australia, Singapore, Guam (USA) and Saipan (USA)]. The division of countries into North and South was based on the climate map proposed by previous research [26]. Most countries affected by JEV are represented in this study, with the exception of Bhutan, Laos, Cambodia, Brunei, the Philippines, North Korea, Pakistan and the islands of the Pacific, due to lack of publications.

Age categories of vertebrate hosts consisted of young, adult, and both. Young cattle were defined as animals aged up to 24 months, pigs up to 7 months old, and sheep and goats up to 14 months old [27]. All remaining host species categories were reported as young or adults, as the data extracted in the systematic review did not include age specification.

When fitting meta-regression models, referent categories of predictors were selected, according to biological plausibility or number of observations.

## Results

### Systematic review of the literature

From 1855 articles initially identified, 171 were selected as relevant and subjected to data extraction and risk of bias assessment. Fifty-nine percent (*n* = 101) of the articles were observational studies, 37% (*n* = 63) were experimental studies, and 4% (*n* = 7) had an experimental and an observational component. About 60% of the articles reported vector competence, contrasting with host competence (29%) and more than one category (11%).

Regarding the risk of bias assessment, all observational studies had a low risk of bias, defined as plausible bias that is unlikely to seriously alter the results, and all experimental studies had a high risk of bias, defined as plausible bias that seriously weakens confidence in the results [20].

Sixty-seven observational studies were considered for the meta-analysis models, 18 reporting proportion of JEV infection in vectors with more than 1% infection and more than 1000 individual mosquitoes; 33 reporting proportion of JEV infection in hosts; and 16 reporting MIR. The remaining 104 articles pertained to other outcome measures that are out of the scope of this manuscript.

### Meta-analyses

Given the difference in magnitude of the outcomes of interest across vectors, a subgroup analysis by mosquito species was performed. Summary effect measures (pooled estimates) and their 95% confidence intervals, both the logit and the back-transformed estimates, expressed as percentages, are presented by mosquito species in Tables 3, 4 and 5. Weights, by mosquito species, are computed using a variation of the inverse-variance approach [24], and are presented as percentage of the overall total.

**Table 3:** Subgroup meta-analysis^a^ of studies reporting proportion of JEV infection in vectors grouped by mosquito species. Each effect size (computed for the group of studies reporting proportion of JEV in each mosquito species) represents pooled estimates (effect size) of the outcome for each mosquito species, and the overall represents the overall pooled estimate across all mosquito species

Mosquito species	Effect size (logit)	95% CI (logit)	Proportion of JEV infection^b^	95% CI (proportion)	% weight
*Aedes vexans*	-1.79	-3.91–0.33	0.14	0.02–0.58	1.29
*Anopheles minimus*	-1.79	-3.91–0.33	0.14	0.02–0.58	1.29
*Anopheles tessellatus*	-1.79	-3.91–0.33	0.14	0.02–0.58	1.29
*Armigeres subalbatus*	-1.85	-3.07– -0.64	0.14	0.04–0.35	1.78
*Culex annulus*	1.35	-4.39–7.10	0.79	0.01–1.00	3.83
*Culex fuscocephala*	1.22	-4.41–6.85	0.77	0.01–1.00	3.76
*Culex tritaeniorhynchus*	-1.04	-1.21– -0.88	0.26	0.23–0.29	28.59
*Culex gelidus*	-0.04	-3.06–2.98	0.49	0.04–0.95	3.37
*Anopheles subpictus*	-1.46	-1.80– -1.13	0.19	0.14–0.24	23.67
*Aedes butleri*	-2.17	-3.21– -1.13	0.10	0.04–0.24	1.87
*Coquilettidia crassipes*	0.69	-1.70–3.08	0.67	0.15–0.96	1.15
*Culex annulirostris*	1.05	0.97–1.13	0.74	0.73–0.76	2.19
*Culex bitaeniorhynchus*	0.85	-0.50–2.20	0.70	0.38–0.90	1.70
*Culex palpalis*	1.08	-0.07–2.24	0.75	0.48–0.90	3.65
*Culex quinquefasciatus*	-0.13	-4.20–3.94	0.47	0.01–0.98	2.61
*Culex sitiens*	-0.51	-1.94–0.92	0.38	0.13–0.72	1.66
*Culex whitmorei*	-1.63	-3.16– -0.10	0.16	0.04–0.47	2.52
*Mansonia septempunctata*	-0.92	-2.08–0.24	0.28	0.11–0.56	1.81
*Ochleratus normanensis*	-0.82	-1.27– -0.37	0.31	0.22–0.41	4.17
*Verrallina funerea*	0.00	-1.96–1.96	0.50	0.12–0.88	1.37
*Culex pipiens fatigans*	4.17	2.19–6.15	0.98	0.90–1.00	1.36
*Aedes albopictus*	0.17	-0.50–0.84	0.54	0.38–0.70	2.05
*Culex pipiens*	-1.39	-3.59–0.81	0.20	0.03–0.69	1.25
*Culex pipiens quinquefasciatus*	-2.04	-3.24– -0.84	0.12	0.04–0.30	1.79
Overall	-0.70	-1.07– -0.33	0.33	0.26–0.42	100

^a^Random-effects meta-analysis using the method of DerSimonian & Laird [24] to estimate the variance between studies, using a restricted maximum likelihood (REML) algorithm. *I* ^2^ range: 36.5% (*P*-value < 0.001) (*Anopheles subpictus*) - 98.6% (*P*-value = 0.64) (*Culex annulus*)

^b^ *p* = (*e* ^*logit*^/ (*e* ^*logit*^+1))

*Abbreviation*: *CI* confidence interval

**Table 4:** Subgroup meta-analysis^a^ of studies reporting the proportion of JEV infection in vertebrate hosts grouped by host species. Each effect size (computed for the group of studies reporting proportion of JEV in each vertebrate host species) represents pooled estimates (effect size) of the outcome for each host species, and the overall represents the overall pooled estimate across all vertebrate host species

Vertebrate host species	Effect size (logit)	95% CI (logit)	Proportion of JEV infection^b^	95% CI (proportion)	% weight
Pigs	-0.36	-0.64– -0.08	0.41	0.35–0.48	59.11
Birds	-2.05	-3.25– -0.84	0.11	0.04–0.30	6.12
Sylvatic mammals	-0.95	-1.90–0.01	0.28	0.13–0.50	4.37
Cattle	-0.25	-1.17–0.67	0.44	0.24–0.66	3.16
Sheep and goats	-0.77	-1.01– -0.53	0.32	0.27–0.37	2.91
Cats and dogs	0.58	-0.40–1.56	0.64	0.40–0.83	2.38
Chickens	-2.47	-2.94– -2.01	0.08	0.05–0.12	2.67
Ducks	-0.67	-2.60–1.26	0.34	0.07–0.78	2.02
Herons	-0.94	-1.25– -0.63	0.28	0.22–0.35	13.97
Horses and donkeys	0.62	-0.24–1.49	0.65	0.44–0.82	0.95
Wild pigs	0.12	-2.93–3.17	0.53	0.05–0.96	0.96
Bats	-3.26	-4.67– -1.85	0.04	0.01–0.14	0.45
Reptiles and amphibians	-1.20	-2.12– -0.29	0.23	0.11–0.43	0.92
Overall	-0.62	-0.83– -0.41	0.35	0.30–0.40	100

^a^ Random-effects meta-analysis using the method of DerSimonian & Laird [24] to estimate the variance between studies, using a restricted maximum likelihood (REML) algorithm. *I* ^2^ range: 60.00% (*P*-value < 0.001) (chickens) - 96.8% (*P*-value = 0.64) (pigs)

^b^ *p* = (*e* ^*logit*^/ (*e* ^*logit*^+1))

**Table 5:** Subgroup meta-analysis^a^ of studies reporting proportion of minimum infection rates (MIR) in vectors grouped by mosquito species. Each effect size (computed for the group of studies reporting proportion of JEV in each mosquito species) represents pooled estimates (effect size) of the outcome for each mosquito species, and the overall represents the overall pooled estimate across all mosquito species

Mosquito species	Effect size (logit)	95% CI (logit)	MIR^b^	95% CI (proportion)	% weight
*Anopheles sinensis*	-1.32	-1.36– -1.28	0.21	0.20–0.22	2.44
*Culex tritaeniorhynchus*	-1.19	-1.70– -0.68	0.23	0.15–0.34	21.22
*Mansonia uniformis*	-1.85	-3.34– -0.36	0.14	0.03–0.41	4.02
*Anopheles subpictus*	0.93	-0.03–1.89	0.72	0.49–0.87	4.38
*Culex gelidus*	-1.01	-1.80– -0.22	0.27	0.14–0.44	15.36
*Culex fuscocephala*	-1.47	-2.53– -0.41	0.19	0.07–0.40	7.23
*Culex vishnui*	-0.37	-0.48– -0.26	0.41	0.38–0.44	4.80
*Culex* spp.	-0.34	-1.07–0.38	0.41	0.26–0.59	18.55
*Culex pseudovishnui*	-1.32	-1.65– -1.00	0.21	0.16–0.27	5.42
*Culex sitiens subgroup*	0.84	-2.67–4.34	0.70	0.07–0.99	7.26
*Ochlerotatus vigilax*	-0.85	-0.93– -0.77	0.30	0.28–0.32	2.43
*Anopheles vagus*	-0.53	-1.16–0.10	0.37	0.24–0.52	2.16
*Aedes* spp.	-1.21	-1.43– -0.99	0.23	0.19–0.27	2.40
*Culex whitmorei*	-1.21	-1.56– -0.86	0.23	0.17–0.30	2.34
Overall	-0.79	-1.06– -0.51	0.31	0.26–0.37	100

^a^Random-effects meta-analysis using the method of DerSimonian & Laird [24] to estimate the variance between studies, using a restricted maximum likelihood (REML) algorithm. *I* ^2^ range: 80.7% (*P*-value = 0.06) (*Anopheles subpictus*) – 100.00% (*P*-value = 0.64) (*Culex sitiens subgroup*)

^b^ *p* = (*e* ^*logit*^/ (*e* ^*logit*^+1))

Subgroup analysis showed large differences (range = 0.10 in *Aedes butleri* to 0.98 in *Culex pipiens fatigans*) between the subgroup overall estimates of the proportion of JEV infection. The lowest proportion of infection was 0.10, meaning that 10% of the total number of *A. butleri* pools tested in the 18 articles included in this meta-analysis were infected with JEV. On the other hand, 98% of the *C. pipiens fatigans* pools were reported to be JEV positive across studies. Although pooled estimates of the proportion of JEV infection in vectors showed some variability, this variability was considered unimportant for mosquito species *Anopheles subpictus* and *Ochleratus normanens* (*I* ^2^ = 36.5% and *I* ^2^ = 37.0%, respectively), and moderate for *C. tritaeniorhynchus* and *Culex palpalis* (*I* ^2^ = 58.2% and *I* ^2^ = 52.8%, respectively). There was evidence of considerable heterogeneity (*I* ^2^ > 85%) for estimates of the proportion of JEV infection in all remaining mosquito species (Table 3).

Subgroup meta-analysis of studies reporting the proportion of JEV infection grouped by vertebrate hosts also ranged greatly, with horses and donkeys showing the highest proportion of JEV infection (0.65) and bats showing the lowest (0.04). Overall, the proportion of JEV infection across all vertebrate host species was 35%. Results of pooled estimates are listed in Table 4.

Furthermore, in these models the variation in the pooled estimates was substantial to considerable: in pigs, birds, and sylvatic mammals, 97% of the variability in the effect size was due to heterogeneity, while in cattle and wild pigs it was 92%. Point estimates in ducks and herons also had considerable heterogeneity (*I* ^2^ = 93.8% and 90.7%, respectively) and chickens, and cats and dogs had substantial heterogeneity (*I* ^2^ = 60.0% and 76.2%, respectively).

Summary effect measures of reported MIR are presented in Table 5 and ranged from 0.14 in *Mansonia uniformis* to 0.72 in *A. subpictus*. Pooled estimates of MIR showed considerable heterogeneity across studies in all mosquito species, with *I* ^2^ varying from 80.7% in *A. subpictus* to 100% in the *Culex sitiens* subgroup.

It is important to emphasize that because of the high heterogeneity in some of the point estimates obtained from meta-analyses models, pooled estimates were provided for reference only.

### Meta-regression

#### Univariable and multivariable meta-regression models

Results of the univariable meta-regression models of the study results on predictors that can further explain variation in effects between studies for the proportion of JEV infection in vectors are presented in Table 6. Predictor variables including mosquito species, diagnostic method, country, and capture method were significant in the univariable screen (*P*-value < 0.1). Predictors pertaining to season and mosquitoes/pool were not significantly associated (*P*-value > 0.1) with the outcome.

**Table 6:** Univariable meta-regression model for the proportion of JEV infection in vectors. Coefficients, *P*-values, and 95% confidence intervals (CI) of the association between predictors of interest with the proportion of JEV infection in vectors (*n* = 18 studies). Random effects meta-regression models use the restricted maximum likelihood method (REML)

Predictor	*n*	Coefficient (logit)	SE (logit)	95% CI (logit)	*P*-value	Overall *P*-value
Mosquito species						0.08
*Culex tritaeniorhynchus*	10	Reference				
*Aedes albopictus*	1	1.27	1.25	-1.27–3.81	0.32	
*Aedes butleri*	1	-1.07	1.32	-3.75–1.61	0.42	
*Aedes vexans*	1	-0.69	1.62	-4.00–2.61	0.67	
*Anopheles minimus*	1	-0.69	1.62	-4.00–2.61	0.67	
*Anopheles subpictus*	1	-0.25	0.48	-1.23–0.74	0.61	
*Anopheles tessellatus*	1	-0.69	1.62	-4.00–2.61	0.67	
*Armigeres subalbatus*	1	-0.75	1.36	-3.51–2.01	0.58	
*Coquillettidia crassipes*	1	1.79	1.72	-1.72–5.29	0.31	
*Culex annulirostris*	1	2.15	1.20	-0.30–4.59	0.08	
*Culex annulus*	2	2.29	0.95	0.37–4.22	0.02	
*Culex bitaeniorhynchus*	1	1.95	1.39	-0.88–4.78	0.17	
*Culex fuscocephala*	2	2.51	0.96	0.56–4.45	0.01	
*Culex gelidus*	2	1.11	1.01	-0.96–3.17	0.28	
*Culex palpalis*	1	2.09	0.97	0.11–4.06	0.04	
*Culex pipiens*	1	-0.29	1.65	-3.65–3.07	0.86	
*Culex pipiens fatigans*	1	5.27	1.58	2.06–8.48	< 0.001	
*Culex pipiens quinquefasciatus*	1	-0.94	1.35	-3.69–1.81	0.49	
*Culex quinquefasciatus*	2	0.96	1.16	-1.41–3.32	0.42	
*Culex sitiens*	1	0.59	1.41	-2.28–3.46	0.68	
*Culex whitmorei*	3	-0.51	1.18	-2.92–1.90	0.67	
*Mansonia septempunctata*	1	0.18	1.34	-2.55–2.91	0.90	
*Ochleratus normanensis*	1	0.16	0.90	-1.68–2.00	0.86	
*Verralina funerea*	1	1.10	1.57	-2.10–4.30	0.49	
Intercept		-1.10	0.32	-1.76– -0.44	< 0.001	
Diagnostic method						0.01
Virus isolation	9	Reference				
Not reported	2	0.41	0.50	-0.60–1.43	0.42	
PCR	4	-1.24	0.55	-2.34– -0.15	0.03	
Antigen-capture enzyme assays	3	-0.98	0.48	-1.95– -0.01	0.05	
Intercept		-0.31	0.32	-0.96–0.33	0.34	
Country						0.01
Australasia	3	Reference				
China and Taiwan	3	0.07	0.55	-1.03–1.16	0.90	
India	5	-1.34	0.50	-2.35– -0.33	0.01	
Japan and South Korea	5	-1.20	0.53	-2.27– -0.13	0.03	
Thailand	2	-2.20	1.09	-4.40– -0.01	0.05	
Intercept		0.02	0.37	-0.73–0.77	0.96	
Capture method						< 0.01
Mechanical visual and olfactory	4	Reference				
Not reported	2	-0.81	0.99	-2.80–1.18	0.42	
Manual active	1	4.46	0.60	3.25–5.67	< 0.001	
Manual and mechanical	3	-0.76	0.32	-1.41– -0.11	0.02	
Manual passive	4	-1.12	0.33	-1.79– -0.46	< 0.001	
Mechanical olfactory	3	-1.01	0.54	-2.10–0.08	0.07	
Mechanical visual	1	-1.32	1.08	-3.50–0.86	0.23	
Intercept		-0.29	0.23	-0.76–0.18	0.22	

*Abbreviation*: *SE* standard error

When compared to *C. tritaeniorhynchus*, a higher proportion of JEV infection was reported in the following species: *Coquillettidia crassipes*, *Culex annulirostris*, *Culex annulus*, *Culex bitaeniorhynchus*, *Culex fuscocephala*, *C. palpalis* and *C. pipiens fatigans* (Table 6). Proportion of JEV infection among mosquitoes was lower when either PCR or antigen-capture enzyme assays were used for diagnosis compared to virus isolation. The proportion of JEV infection in vectors reported in articles from China and Taiwan was higher than from the Australasia region, whereas other countries (India, Japan and South Korea and Thailand) reported lower proportion of JEV infection than Australasia.

Articles reporting the use of the manual active method of mosquito capture revealed greater proportion of JEV infection in vectors compared to articles reporting the use of the mechanical visual and olfactory method. The remaining capture methods (manual and mechanical, manual passive, mechanical olfactory and mechanical visual) had lower reported proportions of JEV infection (Table 6).

Mosquito species, capture method, and country were significant in the univariable screen and thus, were included in a multivariable model. In addition, there was evidence that capture method acted as a confounder of the association between mosquito species, our main predictor of interest, and the outcome. The final model for the proportion of JEV infection in vectors however, consisted of 57 observations [thus < 10 (*k* + 1)], which prevented us from fitting a multivariable model. Therefore, only the results from the univariable analysis meta-regression are provided (Table 6).

A univariable meta-regression screen was performed to determine associations between each of the predictors of interest (host species, region, season, age category and diagnostic method) and the proportion of JEV infection in vertebrate hosts (data not shown). Vertebrate host species, region, and season were significantly associated (*P*-value < 0.1) with the outcome.

The proportion of JEV infection in wild pigs, horses and donkeys, cats and dogs, and cattle was greater compared to domestic pigs. Conversely, all other species (bats, birds, chickens, ducks, herons, reptiles and amphibians, sheep and goats and sylvatic mammals) had lower proportion of JEV infection than pigs. Proportion of infection in vertebrate hosts was greater in the southern region compared to the northern region, and greater in all season categories (all year round, more than one trimester, and trimesters 1, 2, and 4) compared to the third trimester (June-August).

The multivariable meta-regression model of proportion of JEV infection in vertebrate hosts is available in Table 7. Host species and region were significantly associated (*P*-value < 0.1) with the outcome and thus considered in the multivariable meta-regression model. Proportion of JEV infection in wild pigs, horses and donkeys, and cats and dogs was greater compared to domestic pigs. Bats, birds, cattle, chickens, ducks, herons, reptiles and amphibians, sheep and goats and sylvatic mammals had lower proportion of JEV infection than pigs. Moreover, the proportion of infection in vertebrate hosts was greater in the southern region compared to the northern (Table 7).

**Table 7:** Multivariable meta-regression model for the proportion of JEV infection in vertebrate hosts. Coefficients, *P*-values, and 95% confidence intervals (CI) of the association between predictors of interest with the proportion of JEV infection in vertebrate hosts (*n* = 33 studies). Random effects meta-regression models use the restricted maximum likelihood method (REML)

Predictor	*n*	Coefficient (logit)	SE (logit)	95% CI (logit)	*P*-value	Overall *P*-value
Host species						< 0.01
Pigs	21	Reference				
Bats	2	-3.78	1.72	-7.17– -0.40	0.03	
Birds	7	-2.49	0.52	-3.51– -1.46	< 0.001	
Cats and dogs	5	0.07	0.78	-1.47–1.61	0.93	
Cattle	4	-0.77	0.69	-2.14–0.59	0.26	
Chickens	9	-2.82	0.73	-4.26– -1.37	< 0.001	
Ducks	4	-1.18	0.84	-2.84–0.49	0.16	
Herons	5	-0.33	0.35	-1.01–0.36	0.35	
Horses and donkeys	4	0.13	1.20	-2.23–2.50	0.91	
Reptiles and amphibians	2	-0.39	1.21	-2.77–1.99	0.75	
Sheep and goats	5	-0.91	0.70	-2.30–0.48	0.20	
Sylvatic mammals	2	-0.10	0.58	-1.24–1.05	0.87	
Wild pigs	2	1.02	1.19	-1.32–3.36	0.39	
Region						< 0.01
North	16	Reference				
South	17	1.37	4.77	0.80–1.93	< 0.001	
Intercept		-0.85	-4.67	-1.20– -0.49	< 0.001	

*Abbreviation*: *SE* standard error

Results of the univariable meta-regression models of the proportion of MIR in vectors are presented in Table 8. Diagnostic method and capture method were significantly associated (*P*-value < 0.1) with the MIR outcome. Minimum infection rates in mosquitoes were greater in articles reporting the use of PCR and lower in those reporting the use of virus isolation compared to the articles reporting the use of antigen-capture enzyme assays as the method of diagnosis. Lastly, MIR in mosquitoes were greater when the method of capture reported was a combination of manual and mechanical, compared to manual passive, and lower when the method used was either mechanical visual or mechanical olfactory (Table 8). A multivariable meta-regression model of MIR could not be built due to the low number of observations (*n* = 46), hence, the results of univariable models are presented (Table 8).

**Table 8:** Univariable meta-regression model for minimum infection rates (MIR). Coefficients, *P*-values, and 95% confidence intervals (CI) of the association between predictors of interest with minimum infection rates (MIR) in vectors (*n* = 16 studies). Random effects meta-regression models use the restricted maximum likelihood method (REML)

Predictor	*n*	Coefficient (logit)	SE (logit)	95% CI (logit)	*P*-value	Overall *P*-value
Diagnostic method						0.02
Antigen-capture enzyme assays	8	Reference				
PCR	5	0.08	0.78	-1.50–1.65	0.92	
Virus isolation	3	-1.50	0.53	-2.56– -0.43	0.01	
Intercept		-0.34	0.32	-1.00–0.31	0.29	
Capture method						0.07
Manual passive	4	Reference				
Not reported	1	-0.07	0.78	-1.64–1.49	0.93	
Manual and mechanical	3	0.23	0.90	-1.58–2.05	0.80	
Mechanical olfactory	4	-0.44	0.78	-2.01–1.13	0.58	
Mechanical visual	2	-1.66	0.68	-3.04– -0.28	0.02	
Intercept		-0.25	0.52	-1.31–0.81	0.63	

*Abbreviation*: *SE* standard error

## Discussion

To the best of our knowledge, this was the first study that has been performed to quantitatively summarize vector and host competence outcomes pertaining to the proportion of JEV infection in vectors and vertebrate hosts. Similarly, this was the first study performed to evaluate sources of heterogeneity, using a meta-analysis approach. Furthermore, we explored study characteristics thought to influence the effect size as sources of heterogeneity using meta-regression models.

Although pooled estimates did not appropriately summarize the proportion of JEV infection in most mosquito species and in all vertebrate host species assessed, as evidenced by the presence of substantial heterogeneity [14], results of pooled estimates are mainly presented (Tables 3, 4 and 5) for reporting purposes. The statistical assessment of heterogeneity reflects artifactual and real sources of variability, with the former being explained by differences in study design issues as well as other differences across studies [25]. Similarly, it is important to note there is inheritably high clinical heterogeneity in animal studies, and specifically in entomological studies, where real differences in response between populations are expected due to the diversity in biological, ecological and geographical factors, among others, arising from the study of multiple and diverse species. Regardless of the source, evaluation and quantification of causes of heterogeneity allows us to better interpret these pooled mean estimates and their range.

The highest proportions of JEV infection in vectors were reported in *C. pipiens fatigans* (98%), *C. annulus* (79%), *C. fuscocephala* (77%), *C. palpalis* (75%) and *C. annulirostris* (74%), which aligns with our current knowledge of the *Culex* genus being reported as important JEV vectors [3].

Using a meta-analysis approach, we aimed to computing pooled estimates of the proportion of JEV infection among the most likely disease vector species, thus studies reporting 0% infection were not included. However, we still considered species with a very low (> 1%) percentage of infection. Percentages < 1% can indicate potential contamination of the sample; when trapping thousands of mosquitoes, cross-contamination with body parts (e.g. legs) from other competent vector species can produce misclassification of test results (i.e. false positives). This source of error was responsible, as an example, for falsely declaring *Culex quinquefasciatus* as a competent vector species for Zika virus during initial stages of the investigation of disease transmission [7]. In some of the studies, after testing thousands of mosquitoes, only few tested positive, likely indicating very low vector competence or a potential misclassification of test results. For some species with reported low percentages of infection, only tens to hundreds of mosquitoes were sampled, suggesting there are neither competent nor abundant. Species with very low percentages of infection are important if they have high population numbers, but if the latter holds, higher population numbers likely would result in higher number of captured/sampled individuals. The role of mosquito species exhibiting low competence and abundance, as vectors would likely be limited.

Moreover, an additional assumption was that fewer than 1000 mosquitoes would not represent an appropriate sample size to determine infection. Articles describing percentages of infection and the overall total number of mosquitoes tested without describing specific sample sizes were still included.

Despite overall high heterogeneity among vector species for this outcome, *A. subpictus* and *O. normanens*, and *C. tritaeniorhynchus* and *C. palpalis* presented unimportant and moderate heterogeneity, respectively, allowing us to accurately summarize and report those pooled estimates. For instance, proportion of JEV infection in *C. tritaeniorhynchus* (26%) could be used in a risk assessment model to evaluate the risk of introduction of JEV via infected *C. tritaeniorhynchus* in a JEV-free region. Other factors contributing to the heterogeneity observed, besides mosquito species, included the type of diagnostic method used to quantify mosquito infection, country where data were collected, and mosquito capture method used. Surprisingly, articles reporting data on *C. tritaeniorhynchus*, which is considered the most significant JEV vector in Southeastern Asia [2, 4, 5, 16, 17] did not show the highest estimates of proportion of JEV infection. However, it is important to highlight that other studies have pointed to the fact that infection in mosquitoes is not always a direct indicator of risk, mainly because vector abundance, density, age and climate play a major role on arbovirus transmission [21]. In any case, *C. tritaeniorhynchus* presented moderate heterogeneity among studies, contrasting with the considerable heterogeneity reported in most mosquito species. Lower heterogeneity could nevertheless be related to the fact that *C. tritaeniorhynchus* was represented in more articles (*n* = 10) than any other mosquito species, thus increasing the precision of the estimate.

The method of mosquito capture was also an important source of heterogeneity, with the manual active method, which includes the use of sweep or drop nets to catch mosquitoes, being associated with a higher proportion of JEV infection in vectors than the mechanical visual and olfactory method, which use attractants to aid in mosquito trapping. Mosquito capture method as a source of heterogeneity is consistent with previous research [10] that suggests that estimation of the parameters involved in vector competence may differ due to ecological heterogeneity and may be affected by method bias, which translates into a mosquito capture method favoring one species over another [10]. An example of method bias is given by previous work [28] when referring to the underrepresentation of some mosquito species, such as *Ochleratus trivittatus* when using light traps (mechanical visual method) and *Culex pipiens* compared to *Aedes vexans* when using CO_2_-baited light traps (mechanical visual and olfactory method). Moreover, Lord et al. [29] also proposed that sampling design of JEV studies tend to be based on capturing mosquitoes from around cattle sheds at dusk, which influence the observed dominance of *C. tritaeniorhynchus* as the primary JEV vector reported in the literature. Hence, different capture methods may enhance the collection of mosquito species with different competence for JEV (manual active method may favor the collection of species with a higher proportion of JEV infection), thus contributing to the heterogeneity reported.

Regarding pooled estimates of proportion of JEV infection in vertebrate hosts, articles report horses and donkeys (65%), cats and dogs (64%) and wild pigs (53%) among the host species with the highest proportions of infection, and not domestic pigs, as expected due to their role as main JEV reservoir hosts. Nevertheless, the species reported, excluding wild pigs, are dead-end hosts and thus do not play a relevant role on the transmission dynamics of JE and JEV. Wild pigs, on the other hand, are amplifying hosts that do contribute to JEV transmission. According to previous research [8], the northern and central coast of California have a significant wild pig population, which could contribute to transmission and establishment of JEV in the USA, should it be introduced in the country. The same would apply to any region potentially at risk that has a considerable population of wild pigs, even if not having an intense pig production or not having been traditionally associated with backyard pig raising.

It is important to note that the existence of positive bias in the resulting estimates of infection in vectors due to non-reporting of negative results is a possibility and should be taken in consideration when interpreting these results.

When exploring potential sources of heterogeneity of point estimates pertaining to the proportion of JEV in hosts, countries were divided into two main geographical regions (North and South) to reduce the number of categories being analyzed, as opposed to considering each country or group of countries as a unique category, similar to what was done for the outcome pertaining to the proportion of JEV in vectors, in which we grouped countries according to geographical proximity. Previous work [26] demonstrated that there is an association between JEV genotype and climate, further dividing the countries where JEV is present into a northern and a southern region. The geographical distribution suggested by that research [26] was thus followed in this study. Proportion of infection in vertebrate hosts was greater in the South compared to the North, which may be related to the fact that southern countries have an endemic pattern of JEV transmission, as opposed to the epidemic pattern found in the temperate regions of northern Asia. Thus, because JEV transmission is present all year round in the southern countries of Asia, there are increased opportunities for host infection, which may lead to the higher proportion of infection reported in vertebrate hosts.

In addition to region, host species also represented an important source of heterogeneity among studies for the outcome proportion of JEV infection in vertebrate hosts, based on a multivariable meta-regression model.

Although considered the main reservoir host for JEV, pigs were not among the vertebrate host species with the highest proportion of JEV infection. Wild pigs, horses and donkeys, herons, and cats and dogs had higher proportion of JEV infection compared to domestic pigs. This may be due to an intensification of industrial pig farming across Asia [6], which led to a decrease in backyard pig farming, coupled with an increase in biosecurity measures. This is a controversial hypothesis, as previous literature suggests that the industrialization of pig farming did, in fact, enhance the risk of JEV transmission [4]. However, other studies support that.

JEV transmission is possible without the intervention of pigs [17] and that JE also occurs in regions of Bangladesh and India, where pig farming is low compared to other livestock, mainly due to differences in religious practices, as Muslims usually do not eat pork [10]. Moreover, van den Hurk et al. [30], determined that pig relocation did not decrease the risk for JEV transmission to humans in northern Australia, further dismissing the importance of pigs as the main JEV amplifying host in specific regions.

Although the highest proportions of JEV infection in vectors were reported in species of the *Culex* genus, the highest MIR, however, was not reported in the same mosquito species (70% in *C. sitiens* subgroup and 72% in *A. subpictus*). Furthermore, MIR pooled estimates were only available for one mosquito species for which high proportion of JEV infection had been reported (*C. fuscocephala*), and the values were not comparable (77% for the proportion of JEV infection outcome versus 19% for the MIR outcome). MIR is one of the methods, along with the MLE, available to estimate the proportion of infected mosquitoes from pooled samples. MLE is defined by previous authors [21] as the proportion (proportion being a parameter for a binomial distribution) of infected mosquitoes that maximizes the likelihood of *n* pools to be infected, as opposed to MIR, which is the ratio of positive mosquito pools to the total number of pools in the sample. While MIR assumes that there is only one infected individual present in a positive pool, MLE uses an algorithm to consider variations in pool size. In other words, MIR represents the proportion of mosquitoes carrying a particular virus, and, in comparison with the MLE method, estimates the lower bound of the infection rate [31]. Potential disparities between mosquito species deemed to have higher proportion of JEV infection but lower MIR can be explained by the true infection rate, number of pool tested and pool sizes. Disparities in the sample size (number of articles included) of each meta-analysis could also play a role in the differences observed. When infection in the mosquito populations are at high levels, during periods of high transmission, or when pool sizes are large, using MIR underestimates mosquito infections [21, 31]. Therefore, MLE data would be important to more accurately assess infection in mosquito populations.

However, although MLE estimation is considered more robust and accurate than MIR, MIR has been more widely adopted for reporting infection rate (*n* = 16) than MLE. In fact, although gathered in the data extraction step of our systematic review, MLE results were not subjected to a meta-analysis because data resulted from a limited number of articles (*n* = 6) and the analysis could not be carried out. Because of the importance of estimating infection rates of mosquito-borne diseases in disease transmission and in surveillance programs, the body of evidence will benefit from studies reporting infection rates using the MLE method.

Sources of heterogeneity between studies for MIR point estimates included the diagnostic method used and the method of mosquito capture. The fact that other predictors tested were not deemed significant to explain heterogeneity may be due to the small number of studies (*n* = 16) included in the meta-regression analysis for this specific outcome.

The approach used in this study allowed us to obtain estimates of variability of proportion of JEV infection in vectors and vertebrate hosts among the studies from which data were retrieved. Pooled estimates of mosquito species presenting unimportant (*A. subpictus* and *O. normanens*) or moderate (*C. palpalis*) heterogeneity could be due to a smaller number of articles reporting JEV infection for those species. As mentioned above, however, *C. tritaeniorhynchus* was the most represented species across studies, making its estimates useful for using as input parameters in risk assessment models assessing the potential introduction of JEV into currently virus-free regions, including the USA. More studies addressing vector competence in underrepresented mosquito species would therefore improve the precision of estimates, granting more accurate data to be incorporated in such predictive models.

Furthermore, our approach to explore heterogeneity is relevant to understand the sources of variability associated with the predictors and outcomes of interest. Our findings provide useful guidelines when interpreting vector and host infection proportions or prevalence, especially when comparing results from studies that use different study designs. We concluded that mosquito and vertebrate host species, diagnostic method, mosquito capture method and country were the predictors explaining most of the heterogeneity among studies. More specifically, this study led to a better understanding of the influence of certain predictors, such as mosquito capture method or species, in the interpretation of the outcomes. Regarding mosquito species, proportion of JEV infection should be cautiously interpreted, as it does not directly translate into higher transmission risk, as suggested by previous literature [21]. Again, JEV transmission results from a complex interplay of factors, such as environmental and ecological characteristics, as suggested by the high heterogeneity found in this meta-analysis, which should be taken into consideration when interpreting infection proportions for each species.

Another important predictor to consider is mosquito capture method, as different methods may attract different mosquito species, thus biasing infection data towards an over or underrepresentation of certain mosquito species [10]. Mosquitoes may also belong to different stages of development, as oviposition traps collect older mosquitoes that have already blood-fed and laid eggs, while light traps or manual aspiration methods usually collect host seeking mosquitoes. This difference in developmental stages of mosquitoes may also impact the results of mosquito collection.

Lastly, although meta-regression models allowed us to investigate whether specific predictors explained any of the heterogeneity of effects between studies, it is important to note that a *post-hoc* selection of characteristics or predictors that might explain heterogeneity can lead to false positive conclusions [32]. Although no specific protocol was in place to identify appropriate covariates, we believe there was a strong rationale for including diagnostic methodologies and study descriptors as covariates of interest. Granting there may be additional confounders that were not accounted for, the small sample size (number of articles) of most of our models limited our ability to fit multivariable models.

A limitation of this study is related to the large variability reported in the outcome measures of interest that translated into the heterogeneity found across articles and demonstrated in the meta-analysis models. These are related to differences in study methodology, data collection, data reporting and results presentation, as well as geographical distribution and environmental factors inherent to those regions. When initially posing the research question for the systematic review, we aimed at investigating vector competence in North America. Because only a few articles could be retrieved from the databases and journal websites, the research question was expanded to include worldwide estimates, thus leading to high levels of variation described in this study. Similarly, while not imposing any restrictions on study design specifications (including year of publication), neither on the predictors analyzed (mosquito capture method, species and diagnostic method), allowed us to retrieve large amounts of data, it also led to the heterogeneity observed. Variability regarding diagnostic methods in particular were related to the large span of years comprised in all articles retrieved (from 1946 to 2016), which reflects on the technical and scientific improvements that occurred over the 70 years covered in the literature search. Moreover, the grouping of predictors pertaining to study characteristics into meaningful and representative categories was challenging due to the large diversity in methodology observed across studies.

## Conclusions

Despite the challenges posed by the large variability among studies, this meta-analysis provides a quantitative summary of results of multiple studies evaluating JEV infection in mosquitoes and hosts. This quantitative approach to vector and vertebrate host competence expands our understanding of the relative importance of vectors and vertebrate hosts on JEV introduction and transmission, addressing an important knowledge gap identified in the beginning of our study, and thus providing useful data to be used in risk assessment models. These models have application in decision-making processes related to the implementation of strategies aiming at preventing the introduction of emerging vector-borne zoonoses in susceptible regions, such as the USA [10]. Future studies should focus on vector competence of underrepresented mosquito species and countries where data are not available, particularly in regions where JE cases have not been reported but that are flagged as potentially at risk. Lastly, though not easily achievable in observational studies, a higher degree of standardization regarding mosquito trapping and JEV diagnostic methods should be aimed for, as it would help obtaining a more accurate quantification of outcomes, such as the ones assessed in the current study.

## Additional file

Additional file 1: Reference 1. Complete list of search terms and different combinations used for searching the selected databases and journals. **Table S1.** Summary of search results including number of original (*n* = 1137), duplicate (*n* = 680), non-primary research (*n* = 38), and total abstracts searched (*n* = 1855) and selected (*n* = 1405), by database source, for further relevance screening. **Table S2.** Inclusion and exclusion criteria for relevance screening. **Table S3.** Outcome measures documented during data extraction. **Table S4.** Description of criteria, outcomes and identification of key domains for risk of bias assessment in observational studies. **Table S5.** Description of criteria, outcomes and identification of key domains for risk of bias assessment in experimental studies. (DOCX 28 kb)

## Abbreviations

EIA: Enzyme immunoassays
HAI: Hemagglutination inhibition tests
IFA: Indirect immunofluorescence assay
JE: Japanese encephalitis
JEV: Japanese encephalitis virus
MIR: Minimum infection rate
MLE: Maximum likelihood estimator
PCR: Polymerase chain reaction
PRNT: Plaque reduction neutralization test
REML: Restricted maximum likelihood
RT-PCR: Reverse transcription polymerase chain reaction
