# Supplementary material for: Meta-analyses of the proportion of Japanese encephalitis virus infection in vectors and vertebrate hosts
Source: Parasit Vectors. 2017 Sep 7;10:418. doi: 10.1186/s13071-017-2354-7 (PMC5590142; doi:10.1186/s13071-017-2354-7)
Supplement: Additional file 1: — Reference 1. Complete list of search terms and different combinations used for searching the selected databases and journals. Table S1. Summary of search results including number of original (n = 1137), duplicate (n = 680), non-primary research (n = 38), and total abstracts searched (n = 1855) and selected (n = 1405), by database source, for further relevance screening. Table S2. Inclusion and exclusion criteria for relevance screening. Table S3. Outcome measures documented during data extraction. Table S4. Description of criteria, outcomes and identification of key domains for risk of bias assessment in observational studies. Table S5. Description of criteria, outcomes and identification of key domains for risk of bias assessment in experimental studies. (DOCX 28 kb) [file 13071_2017_2354_MOESM1_ESM.docx]

**Supplementary information**

**Reference 1.** Complete list of search terms and different combinations used for searching the selected databases and journals.

**Web of Science**

1. TOPIC: (Japanese) AND TOPIC: (Encephalitis) OR TOPIC: (viral encephalitis) OR TOPIC: (JE) OR TOPIC: (JEV) AND TOPIC: (vector competence) AND TOPIC: (mosquito) AND TOPIC: (host competence) AND TOPIC: (United States) OR TOPIC: (US) OR TOPIC: (USA) OR TOPIC: (North America)

Timespan: All years.

Search language=English

Results: 3,122,242

2. TITLE: (((((((japanese AND encephalitis) OR viral encephalitis) OR JE) OR ((((JEV AND vector competence) AND mosquito) AND host competence) AND United States)) OR USA) OR US) OR North America)

Timespan: All years.

Search language=English

Results: 346,820

3. TOPIC: ((japanese encephalitis OR viral encephalitis OR JE OR JEV) AND vector competence AND mosquito AND host competence AND (United States OR USA OR US OR North America))

Timespan: All years.

Search language=English

Results: 45

4. TOPIC: (japanese encephalitis OR viral encephalitis OR JE OR JEV AND vector competence AND mosquito AND host competence AND United States OR USA OR US OR North America)

Timespan: All years.

Search language=Auto

Results: 3,128,582

5. TOPIC: (((japanese AND encephalitis) OR viral encephalitis OR JE OR JEV) AND vector competence AND mosquito AND host competence AND (United States OR USA OR US OR North America))

Timespan: All years.

Search language=Auto

Results: 45

6. Search 3 without region. TS: ((Japanese encephalitis OR viral encephalitis OR JE OR JEV) AND vector competence AND mosquito AND host competence)

Timespan: All years.

Search language=English

Results: 112

**PubMed**

1. All Fields: ((japanese AND encephalitis) OR viral encephalitis OR JE OR JEV) AND vector competence AND mosquito AND host competence AND (United States OR USA OR US OR North America)

Results: 22

2. All Fields: (Japanese AND encephalitis AND (United States OR US OR USA OR North America) AND vector competence AND mosquitoes AND vector competence)

Results: 0

3. All Fields: (Japanese AND encephalitis AND (United States OR US OR USA OR North America) AND mosquitoes)

Results: 129

4. ((Japanese AND encephalitis) OR (viral AND encephalitis) OR JE OR JEV) AND (United States OR US OR USA OR North America) AND mosquito

Results: 1460

5. Search 1 without Region. All Fields: ((Japanese AND encephalitis) OR viral encephalitis OR JE OR JEV) AND vector competence AND mosquito AND host competence)

Results: 25

6. All Fields: ((Japanese encephalitis virus) OR JE OR JEV) AND vector competence AND mosquito AND host competence)

Results: 1

7. All Fields: ((Japanese encephalitis) OR JE OR JEV) AND vector competence AND mosquito AND host competence)

Results: 1

**Armed Forces Pest Management Board**

1. Find results in AFPMB website with all the words: Japanese Encephalitis

Result: 68

http://www.afpmb.org/content/search-afpmborg

2. Find results in AFPMB website with all the words: Japanese Encephalitis United States mosquito

Result: 49

http://www.afpmb.org/content/search-afpmborg

3. Find results in DWFP publications with all the words: Japanese Encephalitis

Result: 17

http://www.afpmb.org/content/dwfp-publication-search

4. Find results in AFPMB website with all the words: Japanese Encephalitis mosquito vector host competence

Result: 14

http://www.afpmb.org/content/search-afpmborg

**Google Scholar**

1. Find article with all of the worlds in title: Japanese encephalitis

And with at least one of the words in the title: United States US USA North America

Results: 61

http://scholar.google.com/scholar?as_q=Japanese+Encephalitis&as_epq=&as_oq=United+States+USA+US+North+America&as_eq=&as_occt=title&as_sauthors=&as_publication=&as_ylo=&as_yhi=&btnG=&hl=en&as_sdt=1%2C5

2. Find article with all of the worlds in the article: Japanese encephalitis mosquito vector competence host

And with at least one of the words in the article: United States US USA North America

Results: 4400

http://scholar.google.com/scholar?as_q=Japanese+Encephalitis+mosquito+vector+competence+host&as_epq=&as_oq=United+States+USA+US+North+America&as_eq=&as_occt=any&as_sauthors=&as_publication=&as_ylo=&as_yhi=&btnG=&hl=en&as_sdt=1%2C5

3. Find article with all of the worlds in title: Japanese Encephalitis

And with at least one of the words in the title: mosquito, vector, competence, host.

Without patents and without citations

1970-2016

Results: 179

http://scholar.google.com/scholar?q=allintitle%3A+Japanese+Encephalitis+mosquito+OR+vector+OR+competence+OR+host&hl=en&as_sdt=0%2C5&as_vis=1&as_ylo=1970&as_yhi=2016

**The American journal of tropical medicine and hygiene**

1. Searching journal content for Japanese Encephalitis (all words) in title, United States US USA (any words) in title or abstract, and mosquito vector competence host (any words) in full text.

Results: 11

http://www.ajtmh.org/search?submit=yes&pubdate_year=&volume=&firstpage=&doi=&author1=&author2=&title=Japanese+Encephalitis&andorexacttitle=and&titleabstract=United+states+US+USA&andorexacttitleabs=or&fulltext=mosquito+vector+competence+host&andorexactfulltext=or&fmonth=&fyear=&tmonth=&tyear=&format=standard&hits=10&sortspec=relevance&submit=yes&submit=Submit

2. Searching journal content for Japanese Encephalitis viral (any words) in title, United States mosquito (all words) in title or abstract, and vector competence host US USA (any words) in full text.

Results: 7

http://www.ajtmh.org/search?submit=yes&pubdate_year=&volume=&firstpage=&doi=&author1=&author2=&title=Japanese+Encephalitis+viral+&andorexacttitle=or&titleabstract=United+States+mosquito&andorexacttitleabs=and&fulltext=vector+competence+host+US+USA&andorexactfulltext=or&fmonth=&fyear=&tmonth=&tyear=&format=standard&hits=10&sortspec=relevance&submit=yes&submit=Submit

3. Searching journal content for Japanese Encephalitis (all words) in title and vector competence host mosquito (any words) in title or abstract.

Results: 33

http://www.ajtmh.org/search?submit=yes&pubdate_year=&volume=&firstpage=&doi=&author1=&author2=&title=Japanese+Encephalitis&andorexacttitle=and&titleabstract=vector+competence+host+mosquito&andorexacttitleabs=or&fulltext=&andorexactfulltext=and&fmonth=&fyear=&tmonth=&tyear=&format=standard&hits=10&sortspec=relevance&submit=yes&submit=Submit

**Journal of Medical Entomology**

1. For title "Japanese encephalitis viral JE JEV" (match any words) and abstract or title "United States US USA" (match any words) and full text or abstract or title "mosquito vector competence host" (match whole all)

Results: 9

http://jme.oxfordjournals.org/search/title%3AJapanese%2Bencephalitis%2Bviral%2BJE%2BJEV%20title_flags%3Amatch-any%20abstract_title%3AUnited%2BStates%2BUS%2BUSA%20abstract_title_flags%3Amatch-any%20text_abstract_title%3Amosquito%2Bvector%2Bcompetence%2Bhost%20text_abstract_title_flags%3Amatch-all%20numresults%3A10%20sort%3Arelevance-rank%20format_result%3Astandard%20jcode%3Ajmedent

2. For title "Japanese encephalitis" (match all words) and abstract or title "United States US USA" (match any words) and full text or abstract or title "mosquito vector competence host" (match whole all)

Results: 1

http://jme.oxfordjournals.org/search/title%3AJapanese%2Bencephalitis%20title_flags%3Amatch-all%20abstract_title%3AUnited%2BStates%2BUS%2BUSA%20abstract_title_flags%3Amatch-any%20text_abstract_title%3Amosquito%2Bvector%2Bcompetence%2Bhost%20text_abstract_title_flags%3Amatch-all%20numresults%3A10%20sort%3Arelevance-rank%20format_result%3Astandard%20jcode%3Ajmedent

3. For title "Japanese encephalitis JE JEV" (match any words) and abstract or title "mosquito vector competence host" (match any words)

Results: 126

http://jme.oxfordjournals.org/search/title%3AJapanese%2Bencephalitis%2BJE%2BJEV%20title_flags%3Amatch-any%20abstract_title%3Amosquito%2Bvector%2Bcompetence%2Bhost%20abstract_title_flags%3Amatch-any%20numresults%3A10%20sort%3Arelevance-rank%20format_result%3Astandard%20jcode%3Ajmedent

4. For title "Japanese encephalitis" (match all words) and abstract or title "mosquito vector competence host JE JEV" (match any words)

Results: 30

http://jme.oxfordjournals.org/search/title%3AJapanese%2Bencephalitis%20title_flags%3Amatch-all%20abstract_title%3Amosquito%2Bvector%2Bcompetence%2Bhost%2BJE%2BJEV%20abstract_title_flags%3Amatch-any%20numresults%3A10%20sort%3Arelevance-rank%20format_result%3Astandard%20jcode%3Ajmedent

**Journal of the American Mosquito Control Association**

1. ti(Japanese AND encephalitis ) OR ti((viral and encephalitis OR JE OR JEV)) AND (vector competence OR host competence) AND (United States OR US OR USA OR North America)

Results: 1454

http://search.proquest.com/results/D8BCEE4BD8C447DEPQ/1?accountid=11789

2. ti(japanese encephalitis OR viral encephalitis OR JE OR JEV) AND ti((United States OR US OR USA OR North America)) AND ab((Vector competence host OR mosquito))

Results: 3

http://search.proquest.com/results/E56B1C3F6F204756PQ/1?accountid=11789

3. ti(japanese encephalitis OR viral encephalitis OR JE OR JEV) AND ti((United States OR US OR USA OR North America)) AND (Vector competence host OR mosquito)

Results: 10

http://search.proquest.com/results/6D28E55E06494409PQ/1?accountid=11789

4. ti((Japanese AND encephalitis) OR (viral AND encephalitis) OR JE OR JEV) AND ab((vector OR host)) AND all(mosquito)

http://search.proquest.com/results/9F0CB20EA7E44588PQ/1?accountid=11789

Results: 149

**Vector borne and zoonotic diseases**

1. You searched for: [Article title: japanese] AND [[Article title: encephalitis] OR [Article title: viral] OR [Article title: je] OR [Article title: jev]] AND [Article title: united] AND [[Article title: sates] OR [Article title: us] OR [Article title: usa] OR [Article title: north]] AND [Article title: america] AND [All: mosquitos]

Results: 0

2. You searched for: [All: japanese encephalitis] AND [All: united] AND [[All: sates] OR [All: us] OR [All: usa] OR [All: north]] AND [All: america] AND [All: mosquitos]

Results: 77

http://online.liebertpub.com/action/doSearch?field1=AllField&text1=Japanese+Encephalitis+&logicalOpe1=AND&field2=AllField&text2=United+Sates+OR+US+OR+USA+OR+North+America&logicalOpe2=AND&field3=AllField&text3=mosquitos+&search=&history=&AfterYear=&BeforeYear=&sortBy=relevancy&displaySummary=false&pageSize=100

3. You searched for: [Article title: japanese encephalitis] AND [Abstract: united] AND [[Abstract: sates] OR [Abstract: us] OR [Abstract: usa] OR [Abstract: north]] AND [Abstract: america] AND [Abstract: mosquitos]

Results: 1

http://online.liebertpub.com/action/doSearch?field1=Title&text1=Japanese+Encephalitis&logicalOpe1=AND&field2=Abstract&text2=United+Sates+OR+US+OR+USA+OR+North+America&logicalOpe2=AND&field3=Abstract&text3=mosquitos&search=&history=&AfterYear=&BeforeYear=&sortBy=relevancy&displaySummary=false&pageSize=100

4. You searched for: [Article title: japanese encephalitis] AND [[Abstract: mosquito] OR [Abstract: vector] OR [Abstract: host]]

Results: 15

http://online.liebertpub.com/action/doSearch?field1=Title&text1=Japanese+encephalitis&logicalOpe1=AND&field2=Abstract&text2=mosquito+OR+vector+OR+host&logicalOpe2=AND&field3=Abstract&text3=&search=&history=&AfterYear=&BeforeYear=&sortBy=relevancy&displaySummary=false&pageSize=100

**Table S1** Summary of search results including number of original (n=1,137), duplicate (n=680), non-primary research (n=38), and total abstracts searched (n=1,855) and selected (n=1,405), by database source, for further relevance screening.

| **Source** | **Originals**^1^  (1,137) | **Duplicates**^2^  (680) | **Non-primary research**^3^  (38) | **Total**  (1,855) |
| --- | --- | --- | --- | --- |
| Web of Science | 77 | 35 | 0 | 112 |
| PubMed | 93 | 36 | 0 | 129 |
| Armed Forces Pest Management Board | 35 | 11 | 22 | 68 |
| The American Journal of Tropical Medicine and Hygiene | 14 | 19 | 0 | 33 |
| Journal of Medical Entomology | 93 | 33 | 0 | 126 |
| Journal of the American Mosquito Control Association | 71 | 68 | 0 | 139 |
| Vector-Borne and Zoonotic Diseases | 5 | 10 | 0 | 15 |
| Google Scholar | 115 | 65 | 0 | 180 |
| Hand Search | 634 | 403 | 16 | 1,053 |
| Total | 1,137 | 680 | 38 | 1,855 |
| Eliminated | | |  | 450 |
| Selected articles | | |  | 1,405 |

^1^Originals refer to the abstracts identified as unique during the literature search.

^2^Duplicates refer to the repeated abstracts found during the literature search (could be repeated more than once).

^3^Non-primary research refers to abstracts from non-peer reviewed articles, conference proceedings, thesis dissertations, and other non-peer reviewed publications.

|  | **Inclusion** | **Exclusion** |
| --- | --- | --- |
| Language | English | Other than English |
| Time period | No restriction regarding time | - |
| Population | Vectors (mosquitoes, other insects) and/or Hosts (vertebrate^1^ hosts) | Vectors other than insects  Non-vertebrate hosts |
| Study type | Challenge trial (laboratory and field)  Field studies (e.g., trapping, capture)  Observational or experimental studies | Non-primary research (thesis)  [Literature reviews] |
| Outcomes and outcome measures | **Vector AND/OR Host Competence to JEV**  Transmission efficiency  Feeding patterns  Host preference  Infectiousness  Susceptibility to infection  Incubation time  Duration of viremia | Vector and/or host competence for other flaviviruses transmitted by ticks^2^ |
| Type of evidence | Peer-reviewed articles | Non-peer reviewed articles, conference proceedings, thesis dissertations, and other non-peer reviewed publications |
| Location | World-wide | - |

**Table S2** Inclusion and exclusion criteria for relevance screening.

^1^ Vertebrate (with a backbone or spinal column; includes mammals, birds, reptiles, amphibians, and fishes).

^2^ Include other flaviviruses transmitted via mosquitoes (West Nile Virus, St. Louis Encephalitis, Yellow Fever, Dengue fever, Zika virus), but not via ticks (Tick-borne encephalitis, Kyasanur Forest Disease, Alkhurma disease, Omsk hemorrhagic fever).

**Table S3** Outcome measures documented during data extraction.

|  | Vector competence | Host competence |
| --- | --- | --- |
| **Transmission efficiency** | Infection^1^, Dissemination^2^ and transmission^3^ rates | - |
| **Host preference** | Host species preference^4^ | - |
| **Susceptibility to infection** | Proportion of JEV infection^5^ | Proportion of JEV infection^6^ |
|  | Minimum infection rate^7^ | - |
|  | Maximum likelihood estimation^8^ | - |

^1^ Infection rate refers to the sum of individual mosquitoes (or pool of mosquitoes) divided by the total number of mosquitoes (or pools of mosquitoes) tested in experimental studies.

^2^ Dissemination rate refers to the proportion of mosquitoes containing virus in their legs, regardless of their infection status (Golnar et al., 2015).

^3^ Transmission rate refers to the proportion of mosquitoes with a disseminated infection that transmits the virus after refeeding (Golnar et al., 2015).

^4^ Host preference pertains to the host species from which mosquito blood meals originate.

^5^Proportion of JEV infection is the sum of positive mosquito pools divided by the total number of pools tested in observational studies.

^6^ Proportion of positive vertebrate hosts equals the sum of positive samples divided by the sum of samples tested.

^7^ Minimum infection rate (MIR) is defined as the ratio of the number of positive mosquito pools to the total number of mosquitoes in the sample, assuming that only one infected individual is present in a positive pool (Bustamante & Lord, 2010).

^8^ Maximum likelihood estimation (MLE) represents the proportion of infected mosquitoes that maximizes the likelihood of the number of pools of a specific size to be virus positive, where the proportion is the parameter of a binomial distribution (Bustamante & Lord, 2010).

**Table S4** Description of criteria, outcomes and identification of key domains for risk of bias assessment in observational studies.

| OBSERVATIONAL STUDIES | | | |
| --- | --- | --- | --- |
| Criteria | **Description** | **Outcome** | **Notes** |
| 1. Study question* | Is the study question clearly defined? | Yes  No  Not reported | *Not reported*: study question is unclear or not well defined. |
| 1. Study population* | Is the study population properly described? | Yes  No  Partially | **KEY DOMAIN**  *Study population*: vertebrate hosts (age, breed, gender, location) and mosquito populations (age, species, gender) clearly reported.  *Partially*: some information is provided. |
| 1. Inclusion/exclusion criteria* | Are inclusion/exclusion criteria properly described? | Yes  No  Not reported | *Not reported*: criteria are unclear or not well defined. |
| 1. Study period** | Was time/duration (month/year/season) of the study reported? | Yes  No  Partially | **KEY DOMAIN**  *Partially:* some information is provided. |
| 1. Study area** | Was the area (country/region) of the study reported? | Yes  No  Partially | **KEY DOMAIN**  *Partially*: some information is provided. |
| 1. Exposures* | Are exposures clearly defined and reported? | Yes  No  Not defined | *Not defined:* exposures are reported but not clearly defined. |
| 1. Outcomes* | Are outcome measures clearly defined and reported? | Yes  No  Not defined | **KEY DOMAIN**  *Not defined:* outcome measures are reported but not clearly defined. |
| 1. Bias* | Was bias reported and controlled for in the statistical analyses? | Yes  No  Not controlled for | *Not controlled for*: bias is reported but controlling for bias is unclear or not reported. |

*Questions that assess internal validity.

**Questions that assess external validity.

**Table S5** Description of criteria, outcomes and identification of key domains for risk of bias assessment in experimental studies.

| EXPERIMENTAL STUDIES | | | |
| --- | --- | --- | --- |
| Criteria | **Description** | **Outcome** | **Notes** |
| 1. Study question* | Is the study question clearly defined? | Yes  No  Not reported | *Not reported:* study question is unclear or not well defined. |
| 1. Study population* | Is the study population properly described? | Yes  No  Partially | **KEY DOMAIN**  *Study population:* vertebrate hosts (age, breed, gender, location) and mosquito populations (age, species, gender) clearly reported.  *Partially:* some information is provided. |
| 1. Intervention* | Is intervention clearly defined (dose, route, viral strain, incubation period, with details sufficient for assessment and reproducibility)? | Yes  No  Not reported | **KEY DOMAIN**  *Not reported:* information concerning intervention is unclear or not well defined. |
| 1. Experimental conditions (challenge trials)** | Are results generalizable (e.g., infection by oral feeding *vs* intrathoracic in vector studies/  infection by mosquito bite *vs* needle in host studies)? | Yes  No  Not applicable | **-** |
| 1. Experimental setting (controlled trials)** | Are results generalizable (e.g., cage *vs* farm/slaughterhouse)? | Yes  No  Not applicable | **-** |
| 1. Randomization* | Is randomization performed and defined? | Yes  No  Not defined | **KEY DOMAIN**  *Not defined:* evidence that randomization is performed but not clearly defined. |
| 1. Blinding* | Is blinding performed and defined? | Yes  No  Not defined | *Not defined:* evidence of blinding but not clearly defined. |
| 1. Outcomes* | Are outcome measures clearly defined and reported? | Yes  No  Not defined | **KEY DOMAIN**  *Not defined:* outcome measures are reported but not clearly defined. |

*Questions that assess internal validity.

**Questions that assess external validity.
